# Assessment of Carotid Intima-Media Thickness as an Early Marker Of Vascular Damage In Hypertensive Children

**DOI:** 10.5935/abc.20170043

**Published:** 2017-05

**Authors:** Liz Andréa Villela Baroncini, Lucimary de Castro Sylvestre, Camila Varotto Baroncini, Roberto Pecoits Filho

**Affiliations:** 1 Pontifícia Universidade Católica do Paraná (PUCPR), Curitiba, PR - Brazil; 2 Universidade Federal do Paraná (UFPR), Curitiba, PR - Brazil

**Keywords:** Child, Hypertension, Carotid Inima-Media Thickness, Biomarkers

## Abstract

**Background:**

The increased carotid intima-media thickness (CIMT) correlates with the presence of atherosclerosis in adults and describes vascular abnormalities in both hypertensive children and adolescents.

**Objective:**

To assess CIMT as an early marker of atherosclerosis and vascular damage in hypertensive children and adolescents compared with non-hypertensive controls and to evaluate the influence of gender, age, and body mass index (BMI) on CIMT on each group.

**Methods:**

Observational cohort study. A total of 133 hypertensive subjects (male, n = 69; mean age, 10.5 ± 4 years) underwent carotid ultrasound exam for assessment of CIMT. One hundred and twenty-one non-hypertensive subjects (male, n = 64; mean age, 9.8 ± 4.1 years) were selected as controls for gender, age (± 1 year), and BMI (± 10%).

**Results:**

There were no significant difference regarding gender (p = 0.954) and age (p = 0.067) between groups. Hypertensive subjects had higher BMI when compared to control group (p = 0.004), although within the established range of 10%. Subjects in the hypertensive group had higher CIMT values when compared to control group (0.46 ± 0.05 versus 0.42 ± 0.05 mm, respectively, p < 0.001; one-way ANOVA). Carotid IMT values were not significantly influenced by gender, age, and BMI when analyzed in both groups separately (Student's t-test for independent samples). According to the adjusted determination coefficient (R²) only 11.7% of CIMT variations were accounted for by group variations, including age, gender, and BMI.

**Conclusions:**

Carotid intima-media thickness was higher in hypertensive children and adolescents when compared to the control group. The presence of hypertension increased CIMT regardless of age, gender, and BMI.

## Introduction

Atherosclerosis is a complex multifactorial disease that begins early, as evidenced by the presence of cardiovascular risk factors developed by children and adolescents,^1^ and documented by previous studies, which indicate that children and adolescents with obesity, dyslipidemia, high blood pressure, and inadequate glucose metabolism have increased risk of developing atherosclerosis in adulthood.^2^ Additionally, the increased carotid intima-media thickness (CIMT) correlates with the presence of atherosclerosis in adults and describes vascular abnormalities in both hypertensive children and adolescents.^3^ Lande et al.^4^ reported that hypertensive children and adolescents with increased CIMT correlated with more severe hypertension assessed by ambulatory blood pressure monitoring, when compared to a control group. Their findings also showed that CIMT is increased in children with primary hypertension, regardless of the effects of obesity. Also, children with end-stage chronic kidney disease (ESCKD) have significantly increased blood pressure levels and CIMT^5-10^. However, CIMT also increases as a physiological reaction of the vessel when adapting to the age-dependent rise in blood pressure in children and adolescents.^11^ In fact, CIMT changes could reflect non-atherosclerotic and adaptive responses to aging and mechanical stress.^11,12^ CIMT seems to coincide with the normal development of children and increases with age, as it does in adults. The objective of the present study was to assess CIMT as an early marker of atherosclerosis and vascular damage in hypertensive children and adolescents compared with non-hypertensive subjects, controlling for age, gender, and body mass index (BMI) and to evaluate the influence of these variables (gender, age, and BMI) on CIMT in each group.

## Methods

### Patients

We selected 148 consecutive hypertensive children regularly followed at the hypertension outpatient clinic of the Pediatric Nephrology Clinic. All subjects had office systolic and/or diastolic BP ≥ 95^th^ percentile for gender and height on ≥ 3 occasions (office hypertension). Hypertension was confirmed by 24 hour ambulatory blood pressure monitoring (ABPM), defined as average daytime and/or nighttime BP ≥ 95^th^ percentile for gender and height according to the ABPM pediatric norms.^13^ Each child had their height and weight measured at the time of their appointments. Body mass index (BMI) was calculated using the standard formula.^14^ Children were considered overweight or obese when they had BMI ≥ 85^th^ and 95^th^ percentile, respectively, for age and gender.^15,16^ Patients' blood and urine samples were collected between 1 week before and one week after the appointment, for assessment of serum glucose, total cholesterol (TC), high-density lipoprotein cholesterol (HDL-C), low-density lipoprotein cholesterol (LDL-C), triglycerides (TGC), and basal insulin. Subjects were classified as having diabetes when treated for insulin-dependent or non-insulin-dependent diabetes or having elevated fasting glucose levels (≥ 126 mg/dL). The use of lipid-lowering drugs or the presence of TC > 200 mg/dL, HDL-C < 40 mg/dL, LDL-C > 100 mg/dL or TGC > 150 mg/dL was recorded.^17^ Subjects also underwent echocardiogram and electrocardiogram (EKG) exams. Exclusion criteria included children with no blood and urine samples, unconfirmed arterial hypertension, and children with diabetes, dyslipidemia, metabolic syndrome, with ESCKD or any other systemic disease. Children who had both essential and secondary hypertension were included in the study. For the control group, we selected 200 consecutive healthy children and adolescents who underwent echocardiography for assessment of an innocent cardiac murmur referred to the study by a private pediatrician. This population was selected among patients from the private health care system. *Systolic blood pressure* (SBP) *and diastolic blood pressure* (DBP) were measured with appropriate cuff sizes according to arm size in the sitting position twice on both arms after a 5 minute-rest before the echo exam. Control and hypertensive subjects were controlled for gender, age (± 1 year), and BMI (± 10%). Exclusion criteria in the control group were children diagnosed with diabetes, dyslipidemia, hypertension, metabolic syndrome, and any systemic disease, according to information provided by their parents or private pediatrician. Each child had their height, weight, and blood pressure measured at the time of their echocardiogram. Children were not sedated before exams. Children who refused to undergo the ultrasound examination and a proper or complete examination, such as very young children, were excluded from the study. The institutional ethical committee approval was obtained for the study. The legal representative of each child provided written informed consent before examination. Children over 10 years of age also signed a consent form.

#### Blood sample analysis of hypertensive children

Venous blood was collected after overnight fasting. Standard techniques were used to determine serum glucose, TC, HDL-C, LDL-C, TGC, and basal insulin. Information about control children's blood sample analysis was provided solely by their parents and their private pediatrician.

#### Ultrasound measurements

Carotid IMT measurements were made using high-resolution B-mode ultrasonography (Philips Medical Systems' HD11 platform) with a broadband width linear array transducer (L 3-12 MHz). Sonography and readings were conducted by a trained and certified sonographer. The subjects were examined in the supine position with an extended neck and the probe in the antero-lateral position. On longitudinal 2D ultrasound images of the carotid artery, the near wall and the far wall were displayed as 2 echogenic lines (the adventitia and intima), separated by the hypoechoic media. The distance between the leading edge of the first bright line of the far wall (lumen-intima interface) and the leading edge of the second bright line (media-adventitia interface) was defined as the CIMT. For this study, we measured the CIMT on the distal 10 mm of the far wall of both the right and left common carotid artery. After zooming and freezing the image, we manually measured the CIMT using electronic calipers. Five measurements were recorded on each side and the average of these measurements was used for the final CIMT analyses, according to the Brazilian Cardiovascular Imaging Department Task Force for Carotid Ultrasound^18^ and Association for European Paediatric Cardiology.^19^

### Statistical analysis

Quantitative variables were described by mean and standard deviation. Qualitative variables were shown as frequencies and percentages. Kolmogorov-Smirnov test was used to assess the normality of the distribution. The Chi-Square test was used to compare qualitative variables between groups. Quantitative variables were compared using the one-way analysis of variance (ANOVA) model and the least significant difference (LSD) for multiple comparisons. For independent samples, two groups were compared using Student's *t*-test. Pearson's correlation coefficient was used to evaluate the linear association between two quantitative variables. A p-value of < 0.05 indicated statistical significance. The sample size calculation was not performed at the present study, since there are no normative values for CIM in healthy children and adolescents. No systematic random sampling was used. The subjects in both groups were chosen by convenience. Data were analyzed with the SPSS v. 20.0 computer program.

## Results

**Figure 1:**
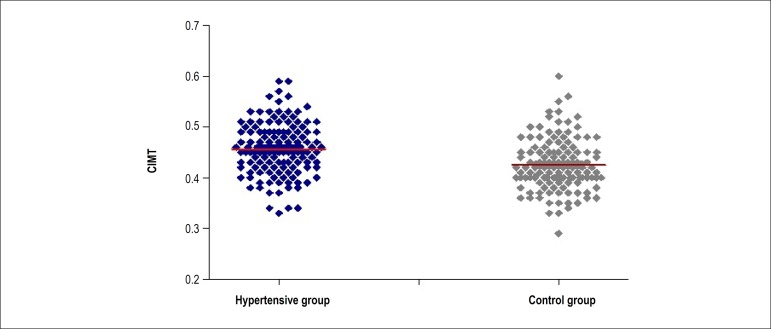
Carotid intima-media thickness (CIMT) values between hypertensive group (HG) and control group (CG).

**Figure 2:**
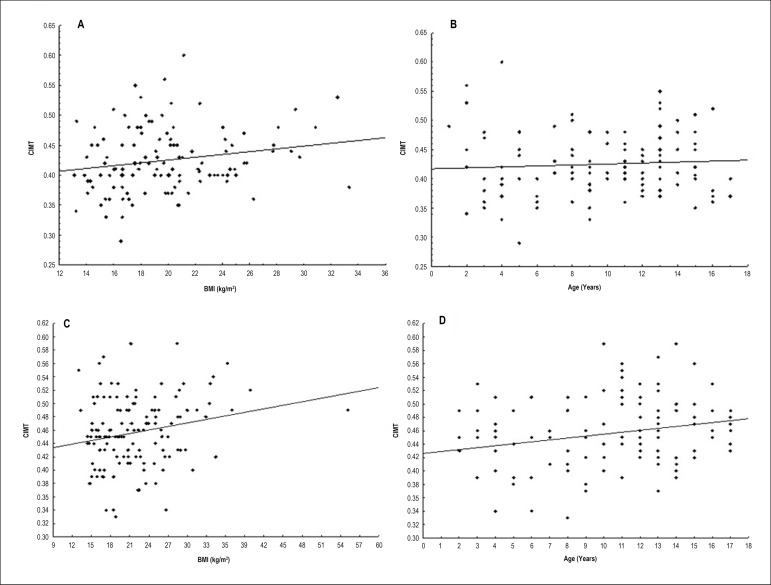
Panels A and B) Correlation between carotid intima-media thickness (CIMT) with age and body mass index (BMI) in hypertensive group.Panels C and D) Correlation between carotid intima-media thickness (CIMT) with age and body mass index (BMI) in control group.

Fifteen hypertensive children and adolescents were excluded from the study for not having undergone lab tests. A total of 133 hypertensive children and adolescents (male, n = 69; mean age, 10.5 ± 4 years) underwent carotid ultrasound exam. All these subjects were undergoing antihypertensive therapy. All hypertensive children and adolescents had normal TC (152 ± 36 mg/dL), normal HDL-C (46 ± 13 mg/dL), normal LDL-C (84 ± 25 mg/dL), normal TGC (86 ± 44 mg/dL), normal fasting glucose (86 ± 10 mg/dL), and normal basal insulin (10 ± 4 mlU/L). The authors identified secondary hypertension in 58 children, of which causes included coartaction of the aorta, reflux nephropathy, ectopic kidney, polycystic kidney disease, chronic pyelonephritis, renal artery stenosis, solitary kidney, and renal atrophy. None of these children were undergoing dialysis treatment. There were no significant differences between children with and without identified secondary hypertension (p = 0,55). None of these children had left ventricular hypertrophy on echocardiogram or EKG alterations. Sixty-four (48%) subjects were within normal BMI range, 33 (24.8%) were considered obese, 33 (24.8%) were considered overweight, and 3 (2.25%) were considered thin. As for the children and adolescents in the control group, 79 subjects were excluded from the study for presenting a metabolic disorder (such as diabetes or dyslipidemia) or any systemic disease according to reported information or because the BMI showed a difference > 10% for age and gender. One hundred and one children and adolescents (males, n = 64; mean age, 9.8 ± 4.1 years) were selected as controls for gender, age, and BMI for the hypertensive group. Sixty-seven (55%) were within normal BMI range, 26 (21%) were obese, 23 (19%) were considered overweight, and 5 (4.1%) were thin. All these subjects had normal echocardiogram results. There were no significant differences regarding gender (p = 0.954) and age (p = 0.067) between groups. Hypertensive subjects had higher BMI when compared to control group (p = 0.004), although within the established range of 10%. Carotid intima-media thickness was higher in hypertensive children when compared to control group (0.46 ± 0.05 versus 0.42 ± 0.05 mm, respectively, p < 0.001; Table 1; Figure 1). Carotid IMT values were not significantly influenced by age, gender, and BMI when analyzed in the 2 groups separately (Figure 2). After multiple linear regression analysis, the increase in CIMT remained independently associated to hypertension (p < 0.001). According to the adjusted determination coefficient (R^2^), only 11.7% of CIMT variations are accounted for by the variations of each group including age, gender, and BMI.

**Table 1:** Basal characteristics of the study population

	CG	HG	p value
**Gender (N/%)**			
Male	64(52.9%)	69 (51.9%)	
Female	57 (47.1%)	64 (48.1%)	0.954
Age (years; mean ± SD)	9.8 ± 4.1	10.5 ± 4	0.162
BMI (kg/m^2^; mean ± SD)	19.9 ± 4.4	21.9 ± 6.3	0.004
CIMT (mm; mean ± SD)	0.42 ± 0.05	0.46 ± 0.05	< 0.001^*^

CG: control group; HG: hypertension group; BMI: body mass index; CIMT: carotid intima-media thickness; SD: standard deviation;

* Student’s t-test for independent samples.

## Discussion

Children with primary hypertension are usually overweight and obese, a fact which makes it difficult to separate the effect of blood pressure from the metabolic disturbances.^20^ According to our findings, 49.6% of the hypertensive children and 40% of the children in the control group were obese or overweight, a factor that would tend to overshadow any potential difference in the groups because of hypertension. However, the present study confirmed that hypertensive children had higher values of CIMT when compared to the control ones regardless of age, gender, and BMI. This finding confirms the studies by Lande et al^4^ and provides evidence that hypertension can lead to vascular abnormalities in childhood, regardless of obesity. Availability of normative CIMT data for children is limited. In the study by Le et al,^21^ child CIMT was compared against the percentile charts available for an ethnicity- and gender-matched 45-year-old adult population to determine the vascular age. They assessed nonobese children with familial dyslipidemia and obese children with multiple atherosclerosis-promoting risk factors such as high triglyceride, high total and LDL cholesterol, low HDL cholesterol, high blood pressure, and high insulin levels. Vascular age was similar in both groups. In the present study, we evaluated only children with hypertension and excluded children with other atherosclerosis-promoting risk factors. CIMT is considered a reflection of multiple risk factors; however, primary contributors to intima-media thickening are age and hypertension.^22-24^ The presence of hypertension significantly increases CIMT values due to the hypertrophy of the media layer of the vessel wall.^25^ Previous studies^26-33^ concluded that a normal carotid arterial wall is unaffected by age or gender until approximately 18 years, after which age, there is diffuse progressive intimal thickening. Hence, in hypertensive children and adolescents, CIMT reflects a physiological reaction of the vessel to adapt the age-dependent rise in blood pressure, plus the effects of hypertension itself. However, hypertension seems to be on the rise with the increase in childhood overweight and obesity. The prevalence of obesity in children is increasing, and thus, inducing an increase in metabolic syndrome of these children. Obesity is associated to several risk factors for cardiovascular disease in adulthood and to other chronic diseases, such as dyslipidemia, hyperinsulinemia, hypertension, and early atherosclerosis.^2,16,20,34-38^ In this regard, any study that aims to evaluate a specific measurement in children and adolescents, such as CIMT, should consider BMI and match this population for gender and age, as performed in the present study.

### Study limitations

The present study has some important limitations identified as (a) inclusion of both essential and secondary hypertension in the hypertensive group; (b) lack of ABPM in the control group and (c) lack of blood samples in the control group. We included children with essential and secondary hypertension. However, we are not certain if the impact of early hypertension, as in secondary causes, will induce higher CIMT in the future when compared to essential hypertension, which usually begins in older children. Moreover, a possible correction of the secondary cause may influence the CIMT measurements. ABPM was not performed in the control subjects to confirm normotension. Finally, we did not request blood and urine samples from the subjects in the control group. These children were selected from the private health care system at a private cardiology clinic and we only obtained information about their blood sample analysis reported by their parents and their private pediatrician.

## Conclusions

Carotid intima-media thickness was higher in hypertensive children and adolescents when compared to the control group. The presence of hypertension increased CIMT regardless of age, gender, and BMI in both hypertensive and non-hypertensive children and adolescents.
